# Supplementary material for: Membrane depolarization mediates both the inhibition of neural activity and cell-type-differences in response to high-frequency stimulation
Source: Commun Biol. 2024 Jun 18;7:734. doi: 10.1038/s42003-024-06359-3 (PMC11189419; doi:10.1038/s42003-024-06359-3)
Supplement: Supplementary file 2 — Supplementary Information [file 42003_2024_6359_MOESM2_ESM.pdf]

SUPPLEMENTARY INFORMATION

Membrane depolarization mediates both the inhibition of neural activity and cell-type-differences in response to high-frequency stimulation.

Jae-Ik Lee[1*], Paul Werginz[1,2], Tatiana Kameneva[3,4], Maesoon Im[5-7], Shelley I. Fried[1,8]

[1]Department of Neurosurgery, Massachusetts General Hospital, Harvard Medical School; Boston, MA, 02114, USA

[2]Institute of Biomedical Electronics, TU Wien, 1040 Vienna, Austria

[3]School of Science, Computing, and Engineering Technologies, Swinburne University of Technology, Hawthorn, VIC 3122, Australia

[4]Department of Biomedical Engineering, University of Melbourne, Parkville, VIC 3010, Australia

[5]Brain Science Institute, Korea Institute of Science and Technology (KIST), Seoul, 02792, Republic of Korea

[6]Division of Bio-Medical Science & Technology, KIST School, University of Science and Technology (UST), Seoul, 02792, Republic of Korea

[7]KHU-KIST Department of Converging Science and Technology, Kyung Hee University, Seoul 02447, Republic of Korea

[8]Boston VA Healthcare System, Rehabilitation, Research and Development, Boston, MA 02130, USA

Supplementary Figure 1: Responses to low-frequency (100 – 300 Hz) stimulation

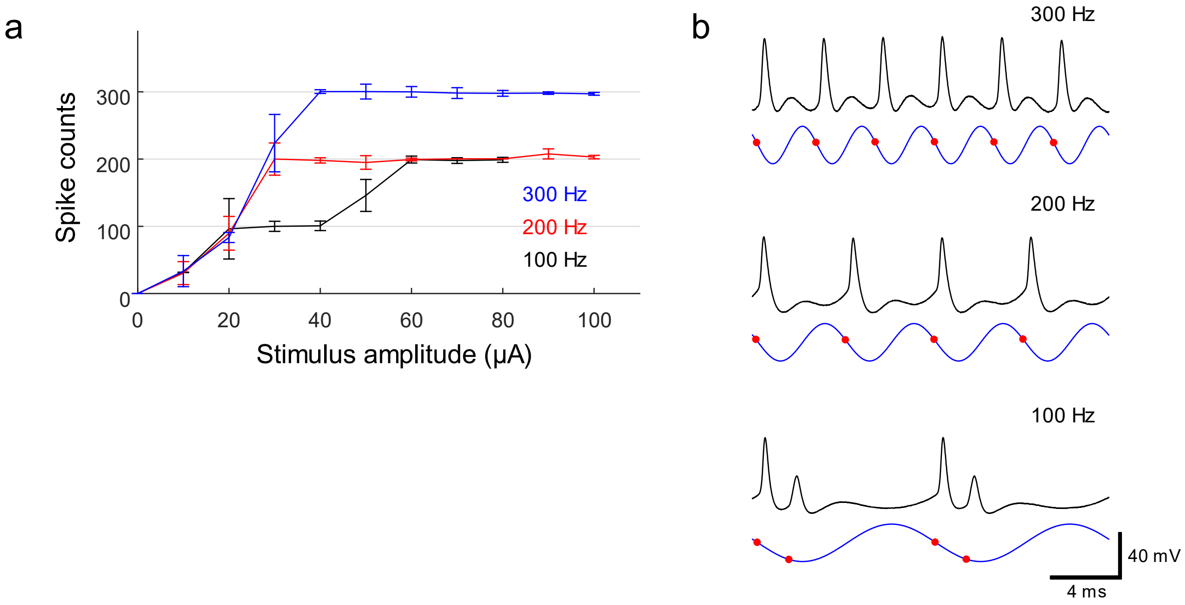

(a) The number of elicited spikes by 100 – 300 Hz stimulation is plotted as a function of stimulus amplitude. **(b)** Action potentials (black traces) and stimulus artifacts (blue traces) extracted from raw recordings. Stimulus amplitude was fixed as 80 μA. Red dots on the stimulus artifacts (i.e., blue traces) indicate the timing of the onset of action potentials.

Supplementary Figure 2: Spiking response patterns are correlated with ΔV_m

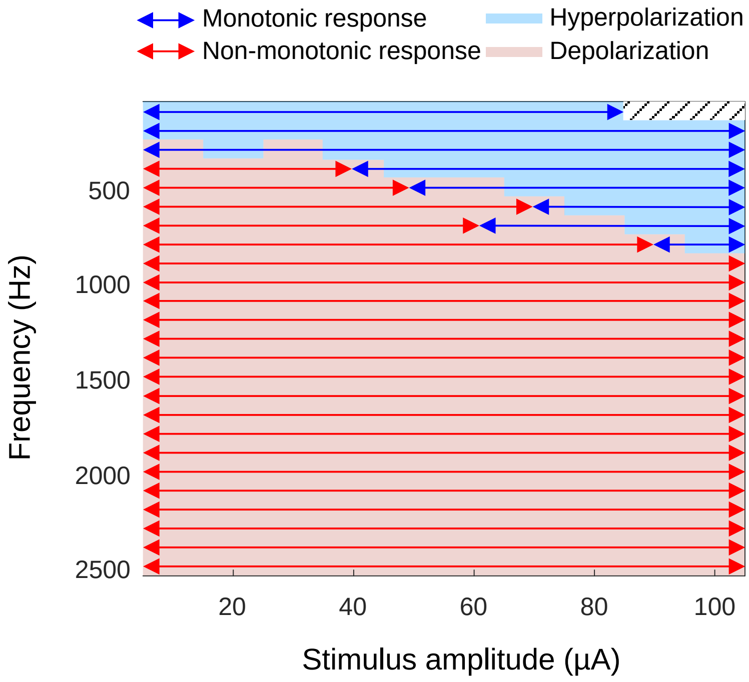

Combinations of stimulus amplitudes and frequencies that hyperpolarized the membrane potential colored red, while those depolarized the membrane potential colored blue. For each frequency, the range of stimulus amplitudes that led to monotonic responses was indicated by two-headed blue arrows, while the amplitudes that led to non-monotonic responses were indicated by red arrows.

Supplementary Figure 3: An example of bifurcation observed in experimental data.

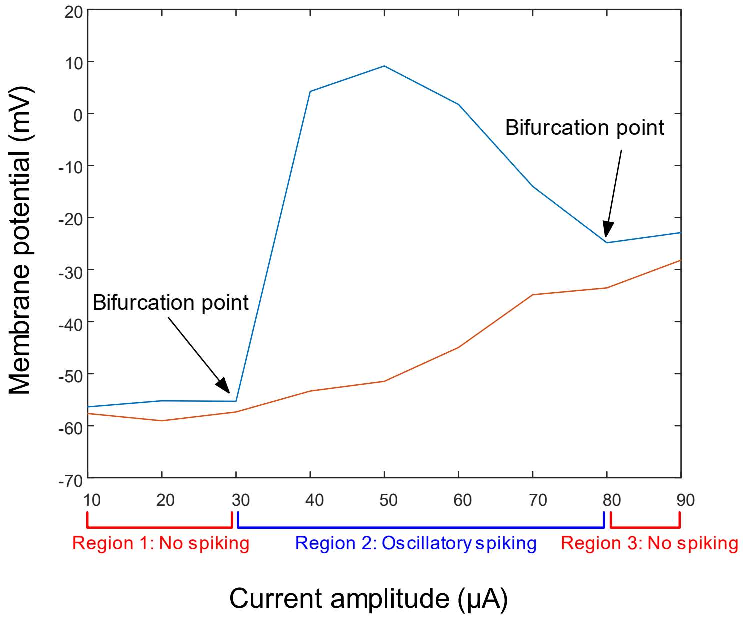

A representative change in the membrane potential to 2,000 Hz stimulation. In this case, the bifurcation parameter is stimulus amplitude and state of the dynamical system is represented as membrane potential. Blue and red traces indicate maximum and minimum of the membrane potential, respectively. Three operating regimes are shown for this neuron: 1) stable no spiking region, 2) repetitive firing region, and 3) no spiking region for high amplitude stimulation. In contrast to the simplified mathematical model bifurcation plots, this figure has two lines for maximum and minimum of the membrane potential in regions 1 and 2 due to fluctuations of the membrane potential in non-spiking regimes.

Supplementary Figure 4: Responses of ON vs. OFF RGCs to 500 Hz stimulation.

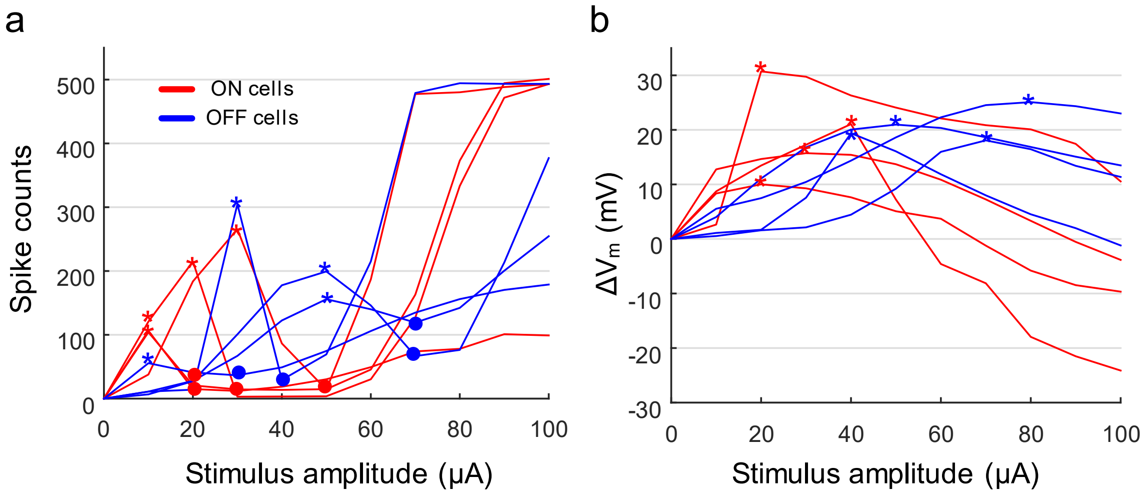

(a) The number of spikes elicited in ON (*n* = 4) and OFF (*n* = 4) cells as a function of stimulus amplitude for a stimulus frequency of 500 Hz (over a duration of 1 second). The asterisks indicate the peak spike counts within the non-monotonic portion of the response, and the circles indicate the transition point from non-monotonic to monotonic responses. **(b)** For the same dataset used in (a), the induced change in membrane potential, ΔV_m, is plotted as a function of stimulus amplitude. The asterisks indicate the peak depolarization levels.
