# Supplementary material for: Membrane depolarization mediates both the inhibition of neural activity and cell-type-differences in response to high-frequency stimulation
Source: Commun Biol. 2024 Jun 18;7:734. doi: 10.1038/s42003-024-06359-3 (PMC11189419; doi:10.1038/s42003-024-06359-3)
Supplement: Supplementary file 3 — Reporting Summary [file 42003_2024_6359_MOESM3_ESM.pdf]

nature portfolio

Corresponding author(s): Jae-Ik Lee

Last updated by author(s): Apr 18, 2024

Reporting Summary

Nature Portfolio wishes to improve the reproducibility of the work that we publish. This form provides structure for consistency and transparency in reporting. For further information on Nature Portfolio policies, see our Editorial Policies and the Editorial Policy Checklist.

Statistics

For all statistical analyses, confirm that the following items are present in the figure legend, table legend, main text, or Methods section.

n/a	Confirmed	
☐	☒	The exact sample size (*n*) for each experimental group/condition, given as a discrete number and unit of measurement
☐	☒	A statement on whether measurements were taken from distinct samples or whether the same sample was measured repeatedly
☐	☒	The statistical test(s) used AND whether they are one- or two-sided<br>*Only common tests should be described solely by name; describe more complex techniques in the Methods section.*
☐	☒	A description of all covariates tested
☐	☒	A description of any assumptions or corrections, such as tests of normality and adjustment for multiple comparisons
☐	☒	A full description of the statistical parameters including central tendency (e.g. means) or other basic estimates (e.g. regression coefficient) AND variation (e.g. standard deviation) or associated estimates of uncertainty (e.g. confidence intervals)
☐	☒	For null hypothesis testing, the test statistic (e.g. *F*, *t*, *r*) with confidence intervals, effect sizes, degrees of freedom and *P* value noted<br>*Give P values as exact values whenever suitable.*
☒	☐	For Bayesian analysis, information on the choice of priors and Markov chain Monte Carlo settings
☒	☐	For hierarchical and complex designs, identification of the appropriate level for tests and full reporting of outcomes
☐	☒	Estimates of effect sizes (e.g. Cohen's *d*, Pearson's *r*), indicating how they were calculated

Our web collection on statistics for biologists contains articles on many of the points above.

Software and code

Policy information about availability of computer code

Data collection	LabVIEW (version 7)
Data analysis	MATLAB scripts (version: R2018a)

For manuscripts utilizing custom algorithms or software that are central to the research but not yet described in published literature, software must be made available to editors and reviewers. We strongly encourage code deposition in a community repository (e.g. GitHub). See the Nature Portfolio guidelines for submitting code & software for further information.

Data

Policy information about availability of data

All manuscripts must include a data availability statement. This statement should provide the following information, where applicable:

- Accession codes, unique identifiers, or web links for publicly available datasets
- A description of any restrictions on data availability
- For clinical datasets or third party data, please ensure that the statement adheres to our policy

The source data and codes are shared in a public repository (https://doi.org/10.17605/osf.io/hnpq4).

Research involving human participants, their data, or biological material

Policy information about studies with human participants or human data. See also policy information about sex, gender (identity/presentation), and sexual orientation and race, ethnicity and racism.

Reporting on sex and gender	N/A; No human subjects were involved in this study
Reporting on race, ethnicity, or other socially relevant groupings	N/A
Population characteristics	N/A
Recruitment	N/A
Ethics oversight	N/A

Note that full information on the approval of the study protocol must also be provided in the manuscript.

Field-specific reporting

Please select the one below that is the best fit for your research. If you are not sure, read the appropriate sections before making your selection.

☒ Life sciences ☐ Behavioural & social sciences ☐ Ecological, evolutionary & environmental sciences

For a reference copy of the document with all sections, see nature.com/documents/nr-reporting-summary-flat.pdf

Life sciences study design

All studies must disclose on these points even when the disclosure is negative.

Sample size	The sample size was determined based (1) on our previous (substantial) experience with electric stimulation of retinal ganglion cells (RGCs), as well as by (2) standards established by other publications studying neuronal activities in RGCs with whole-cell patch recordings. Based on these norms, we acquired data from at least 8 cell for each type. This information is detailed in Results each time a new set of data or analysis is introduced, as well as in the stimulation section of Methods.
Data exclusions	No data was excluded from the analyses.
Replication	All attempts at replication were successful.
Randomization	We recorded neuronal responses of ON and OFF alpha sustained cells from the temporal retina. Alpha RGCs were targeted based on their large somata (>20 μm), and then further classified by their responses to light stimuli. Light stimuli were projected onto the outer segments of the photoreceptors from below using an LCD projector. Stationary flashes with diameters ranging from 100 to 1,000 μm were presented for 1 second. Only cells showing consistent ON or OFF sustained responses across the full range of spot sizes were targeted for further analysis. This information is detailed in Electrophysiology and Light Response section of Methods. Randomization was not relevant for this study as we targeted specific cell types.
Blinding	Blinding was not relevant for this study as we targeted specific cell types

Reporting for specific materials, systems and methods

We require information from authors about some types of materials, experimental systems and methods used in many studies. Here, indicate whether each material, system or method listed is relevant to your study. If you are not sure if a list item applies to your research, read the appropriate section before selecting a response.

Materials & experimental systems

n/a	Involved in the study	
☒	☐	Antibodies
☒	☐	Eukaryotic cell lines
☒	☐	Palaeontology and archaeology
☐	☒	Animals and other organisms
☒	☐	Clinical data
☒	☐	Dual use research of concern
☒	☐	Plants

Methods

n/a	Involved in the study	
☒	☐	ChIP-seq
☒	☐	Flow cytometry
☒	☐	MRI-based neuroimaging

Animals and other research organisms

Policy information about studies involving animals; ARRIVE guidelines recommended for reporting animal research, and Sex and Gender in Research

Laboratory animals	Wild-type C57BL/6J mice were sacrificed at the postnatal days ranging from 28 to 56 for this study.
Wild animals	Animals were purchased from the Jackson Laboratory (Bar Harbor, ME, USA)
Reporting on sex	For this study, both male and female subjects were included, but no sex-based analyses were conducted since there were no reported variations in RGC responses based on sex.
Field-collected samples	This study did not involve samples collected from field.
Ethics oversight	The care and use of animals followed all federal and institutional guidelines and all protocols were approved by the Institutional Animal Care and Use Committee (IACUC) of the Massachusetts General Hospital.

Note that full information on the approval of the study protocol must also be provided in the manuscript.

Plants

Seed stocks	N/A
Novel plant genotypes	N/A
Authentication	N/A
